# Community groups as ‘critical enablers’ of the HIV response in Zimbabwe

**DOI:** 10.1186/1472-6963-13-195

**Published:** 2013-05-26

**Authors:** Morten Skovdal, Sitholubuhle Magutshwa-Zitha, Catherine Campbell, Constance Nyamukapa, Simon Gregson

**Affiliations:** 1 Department of Health Promotion and Development, University of Bergen, Bergen, Norway; 2 Biomedical Research and Training Institute, Harare, Zimbabwe; 3 Institute of Social Psychology, London School of Economics and Political Science, London, UK; 4 Department of Infectious Disease Epidemiology, Imperial College London, London, UK

**Keywords:** Social capital, HIV and AIDS, Community mobilisation, Community groups, Zimbabwe

## Abstract

**Background:**

The Investment Framework for a more effective HIV response has become integral to discussions on how best to respond to the HIV epidemic. The Framework calls for greater synergy and attention to factors that serve as ‘critical enablers’ and optimise HIV programmes. In this paper we argue for recognition of informal and indigenous community groups as ‘critical enablers’ of the HIV response.

**Methods:**

This qualitative study was conducted in Matobo district of the Matabeleland South province in Zimbabwe. It draws on 19 individual in-depth interviews and 9 focus group discussions conducted by local researchers in September and October 2011. Data was thematically analysed.

**Results:**

Four core themes highlight the possibilities and limitations of community groups in the HIV response: (i) Membership of indigenous community groups and group-based dialogue were found to encourage group members to engage with HIV prevention, mitigation and care efforts; (ii) local networks and partnerships between groups and NGOs were said to play an important role in accessing much needed resources to aid indigenous coping with AIDS; (iii) community strengths and resources were recognised and drawn upon in the community group response; (iv) frequent droughts, poverty and stigma served as obstacles to an effective HIV response.

**Conclusions:**

In this context, social groups, although to varying degrees and in direct or indirect ways, play a key role in the HIV response. This suggest that community groups and networks can indeed act as ‘critical enablers’ to the HIV response, and that efforts need to be made to facilitate the contributions of already existing indigenous responses. Local community groups are developing local and collective solutions to structural problems, often independently of external NGO or health service efforts, and begging for synergy and collaboration between local community groups and networks, the health services and other external HIV service delivery sectors.

## Background

In 2011 the Lancet published a policy paper presenting an Investment Framework for a more effective HIV response [1]. The Framework has been endorsed by UNAIDS [2] and calls for more cost effective and strategic use of scarce resources in basic HIV programming. This includes promoting synergy between health and development sectors as well as recognition and facilitation of ‘critical enablers’ for a more effective HIV response. Critical enablers are defined as activities that are necessary to support the effectiveness and efficiency of basic HIV programmes [1,2]. Amongst other activities, the Framework recognizes the importance of the community response to HIV, and promotes community mobilization and community-driven engagement activities as complementary strategies to basic HIV programming.

This recognition is welcomed by practitioners [3,4] and researchers alike who have long argued that a social landscape characterised by active community groups is key to the fight against HIV and AIDS [5-8] and in the caregiving of those affected [9-11]. Nyambedha and Aagaard-Hansen [12] for example have found the HIV epidemic, and the accompanying influx of non-governmental organisations (NGOs) in western Kenya, to have sparked a growth in the number of locally-initiated community groups active in the region. They argue that in a response to HIV and the desire of NGOs to work within local structures, community members have grouped together and used community-based organisations as a platform to re-invent and enact a traditional social system (Duol) that promotes unity, solidarity and an ethics of care and assistance [12].

Our own research in eastern Zimbabwe has explored the role of community groups and networks in the HIV response, focusing on formal grassroots group memberships such as the church, women’s groups, rotating credit associations and so on. Here we have found that group membership have a protective effect against HIV for women (but not for men) [5] as well as contributing to stigma reduction and faster uptake of certain HIV services [13]. More recently we found multiple group membership, as well as groups that provide women with the opportunity to discuss HIV, to protect women against HIV [14]. We have identified several mechanisms through which community groups might exert a positive effect on HIV prevention, care, treatment and impact mitigation. These include group-based opportunities for critical dialogue, facilitating a renegotiation of harmful social norms, sharing of experiences, and the formulation of social action plans and solidarity [15,16]. However, effects were not always positive, cautioning us against over-hasty conclusions about the positive potential of community groups [15]. In some situations, community groups were found, through dialogue, to nurture inaccurate HIV information and boost damaging social norms (ibid.).

The Investment Framework has provided us with a platform to rejuvenate interest in the community response to HIV. Against this background, we examine the ways in which local community group membership facilitates (or hinders) the development of a context where community members can work collaboratively, and with non-governmental organisations (NGOs), to achieve optimal prevention, care and treatment – including behaviour change, care of the sick, acceptance, kindness (vs. stigma) and practical support and assistance for the affected. In doing so, we hope to unpack the different ways community groups act as ‘critical enablers’ of the HIV response.

## Methods

This qualitative study forms part of an on-going study which was granted ethical approval from the Medical Research Council of Zimbabwe (A/681) and Imperial College London (ICREC_9_3_13). Informed written consent was gathered from all research participants on the condition that their identity would not be revealed. Pseudonyms have been used throughout this report.

### Study area and sampling

Matobo is rural and has a population of 110 000 people. It is estimated that 48% of Matobo residents live in abject poverty and between 20-30% are food insecure [17]. The HIV prevalence rate amongst women attending antenatal care at District clinics is 20% [18]. The District has managed to enrol 3 623 people living with HIV or AIDS onto antiretroviral therapy and has identified a total of 9 600 orphaned children – the majority of whom have been orphaned by AIDS and AIDS-related illnesses (ibid.). Typical to the Matabeleland South Province, much of Matobo is arid, making cattle and goat keeping the primary source of income for residents in northern parts of the District. The south of the District has greater opportunities for small-scale and subsistence farming. The District borders South Africa to the south and Botswana to the west, whose industry, cash crop farming and mining companies attract a significant number of Matobo men to look for work. While this enables the transfer of much needed funds to Matobo District, the migration of spouses presents serious challenges to HIV prevention, mitigation, treatment and care services, with some men discontinuing their treatment, and with husbands and wives having different levels of exposure of HIV services available to them.

Matobo District currently has 19 international (e.g., Save the Children, Mildmay, Red Cross and World Vision) and local (e.g., Maranatha, Sikhethimpilo and Jairos Jiri) organisations that are collaborating with community members and groups. In addition to HIV work, many of these organisations also attend to the water and food shortages experienced by the people of Matobo.

A total of 90 community group members participated in this study through focus group discussions and individual in-depth interviews. They were recruited from 9 different community groups, which were purposefully sampled to represent maximum variation (i.e., different types of community groups active in Matobo). The participants were sampled based on their interest to participate and availability. The recruitment of participants was done by researchers from the Biomedical Research and Training Institute in consultation with community guides and a representative from the District AIDS Action Committee based on. Each focus group discussion was made up of members from a social group. Group members with a leadership role were invited to participate in in-depth interviews. The social groups participating in this study include a church group, AIDS support group, burial society, rotating credit society, a women’s group, sports club, youth group, co-operative and a farmer’s group (see Table 1). All participants were over the age of 18.

**Table 1:** Community groups in Manicaland, eastern Zimbabwe

**Group**	**Description**
Church group	Members from the same congregation meet outside of regular church worship times. Engage in Bible study, discussing marital issues, and community outreach, particularly helping families in need (such as those with sick members or orphaned children)
AIDS support group	Loose term to apply to variety of groups including Post HIV test clubs (mostly PLWHA), HIV/ART support groups often organized by clinics, youth groups, peer education groups, home based care groups (members go house to house helping families with sick relatives - doing chores, bathing the sick, sometimes collecting pills from clinic, etc.)
Burial society	Members contribute small sums of money to central fund to cover basic funeral expenses of themselves and other members. Members commit to organizing proper burials for one another and often sing at funerals. Generally meet monthly.
Rotating credit society	Members contribute to central fund and when they reach a certain amount the money is shared for income generating projects such as buying seeds. Members borrow at same interest rate and loans can be made to non-members at a higher rate.
Women's group	Often linked to government women's empowerment initiatives. Supported by government income generating grants.
Sports club	Male dominated. Organize tournaments against other regions. Primarily soccer.
Youth group	Often organized by political parties or teachers, these seek to develop leadership skills and provide recreation for youth (often into 20s – ‘end of youth’ often determined by marriage)
Co-operative	Group members come together to set up an income generating project, co-owned and run by members. The groups sometimes get assistance from NGOs to expand their work.
Farmer's group	Farmers, both male and female, meet monthly to plant crops, discuss weather patterns and new technologies, share labour and access NGO assistance (e.g. for farming implements or water irrigation)

### Data collection and analysis

From each of the 9 different social groups participating in this study, we interviewed group members through in-depth interviews (IDIs) and focus group discussions (FGDs) (see Table 2). The individual and group interviews lasted approximately 90 and 120 minutes respectively and were guided by a topic guide exploring the role of indigenous community groups in the HIV response. Questions covered topics such as the impact of HIV, community openness about HIV, community strategies to support people living with HIV, the importance of community groups in supporting people living with or affected by HIV, local strengths and resources that facilitate a HIV response and the role of networks and partnerships in enhancing the HIV response. The same topic guide was used for both data collection methods, however different probing techniques were applied. Probing questions in the focus group discussions sought to spark debate rather than encouraging participants to answer questions in turn. Experienced Ndebele-speaking fieldworkers conducted the interviews and focus group discussions. Participants were given two bars of soap as a token of appreciation, as well as lunch and reimbursement of transport costs.

**Table 2:** Study participants and methods

**Type of informants**	**IDIs**	**FGDs**	**Total**
AIDS support group members	2 females, 1 male	1 (8 females and 1 male)	12
Burial society group members	2 females	1 (4 females and 2 males)	8
Church group members	1 female, 1 male	1 (11 females)	13
Cooperative members	1 female	1 (7 females and 1 male)	9
Farmers group members	2 females, 1 male	1 (4 females and 5 males)	12
Savings and lending group members	3 females	1 (5 females and 1 male)	9
Soccer club members	3 males	1 (8 males)	11
Women’s group members	2 females	1 (4 females)	6
Youth group members		1 (5 females and 5 males)	10
**Total no. of participants**	19	9 (71 participants)	90

Transcripts were translated from Ndebele to English and imported into Atlas.Ti, a qualitative software package designed for qualitative data analysis. Coding was done in an iterative process using both pre-existing categories and emerging categories. Codes were initially clustered into basic themes, which were subsequently clustered into organising themes and global themes. The thematic network of findings emerging from this qualitative analysis can be found in Table 3.

**Table 3:** Thematic network of findings

**Basic themes**	**Organising themes**	**Global themes**
-Community groups are built on egalitarian principles	Community groups are often characterised by **‘female traits’**	**Group membership and dialogue** encourage members to engage with HIV prevention, mitigation and care efforts
-More women than men take an active role in community groups		
-Men can benefit from joining mixed gender groups		
-Community groups provide members with opportunities for psychosocial development	Community groups are an important source of **support and empowerment** for group members	
-Community groups provide members with a source of support during times of hardship		
-Many community groups, but not all, are committed to HIV work	Many community groups are active in **HIV management**	
-Many community groups contribute to the delivery of HIV services		
-There is a need for externally resourced organisations because of limits of local support structures	**Externally resourced organisations** are important actors in support of HIV-affected community members	**Networks and partnerships** mobilise and make available much needed resources for the community response to HIV prevention, mitigation and care
-NGOs and other externally resourced organisations and active in supporting programmes for HIV-affected community members		
-There is a call for greater NGO support and presence as the demand exceeds supply		
-Community members realise that only by working together can they respond to the HIV epidemic	**Community initiated groups** continue to play a key role in responding to HIV – serving as the entry point for NGO support	
-Groups and active participants are more likely to collaborate with NGOs and contribute with the delivery of HIV services		
-The donor-beneficiary relationship is negotiated carefully for a good fit.	How **NGOs engage** with communities and community groups matters	
-NGOs are thought to have a simplistic understanding of local needs		
-Improved HIV services have changed the social landscape regarding HIV	There has been a **normalisation of HIV and AIDS**	**Community strengths and resources** are recognised and drawn upon in the community response to HIV prevention, mitigation and care
-A gradual openness around HIV has contributed to the slow breakdown of stigmatising attitudes and health damaging practices		
-It is a social norm to provide care and support for vulnerable community members	Community contexts are characterised by an **ethic of care and assistance**	
-Those close to families affected by HIV play a key role in the provision of care and support		
-Some community members support HIV-affected households		
-The lack of rain water and alternative water sources leave many people without enough food	The **natural habitat** is inhospitable, making subsistence farming difficult	**Obstacles and barriers** are acknowledged and considered in responses to HIV
-Poverty makes it difficult for caregivers of vulnerable children to adequately care for them	**Poverty** undermines the well-being of, and responses to, HIV-affected and infected community members	
-The quality and access to public services occasionally prevent HIV-affected community members from accessing support	**Structurally disabling environment** inhibit support for HIV-affected community members	
-Some macro-level influences inhibit a conducive environment for HIV-affected community members		
-Damaging cultural practices and idle talk can still serve as a barrier to HIV management	There continues to be **symbolic and cultural barriers** to the support of HIV-affected community members	

## Results

The social groups participating in this study were formed for a variety of purposes and some (the burial society and the farmers’ association) did not specifically seek to mitigate the impact of HIV for their members. But even these groups provided support in indirect ways. For example the burial society provided its members with the insurance of burial support following the bereavement of self or a close family member. The farmers’ association provided members with the skills to farm as well as access to farming implements from NGOs, opening up opportunities for income generation in a community where poverty dramatically exacerbated the impacts of AIDS. This suggests that even if groups do not mention HIV and do not have HIV related group goals, they can still support those affected. Taking this broad approach we now examine the social dynamics and processes that characterise the role of community groups in the HIV response. To do this we draw on the structure of Table 3, with global themes representing the forthcoming sub-sections. We start off by asking: In what ways might group memberships and dialogue encourage community members to respond to the HIV epidemic?

### Group membership and dialogue

Interview analysis suggested that the dialogue that takes place within community groups is i) often characterised by what one respondent referred to as ‘female traits’; ii) enables support and empowerment of group members, and iii) facilitates the active involvement of community group members in the management of HIV. Each of these points is expanded on below.

Most of the groups, with the exception of the farmers’ association and a soccer club, were predominantly made up of female members. Unsurprisingly our informants tended to characterise groups responding to HIV as female spaces, where values of care dominated and an openness to discuss HIV prevailed, with caring and openness regarded as stereotypically female traits.

“When we look at the issue of HIV, women are the ones who carry the burden. Most of the time it is the women who join groups. For men, unless you tell them that there will be a party with beer, they won’t come. If you tell them the group cares for people living with HIV, it will be like you are alienating them. Usually, really it is the women that participate more in those activities.” Youth group member (male)

It was generally agreed that men fear HIV, which may help explain their lack of participation in groups that embrace and talk about HIV.

“If you look at the composition of home-based caregivers for example, you will not find a male figure among them. It would be better if men were to step forward and spread the HIV awareness message. It is well that the disease is on the decline because of the pills [ART] but the lack of participation by the men is impacting negatively on the situation.” Women’s group member

However it was generally agreed, as articulated by one female AIDS support group member that “by joining a group, men get courage to face the future.” Although the community groups participating in this study had different group membership criteria, a number of participants emphasised the inclusive nature of their groups. Members of a rotating credit and savings association, for example, said it was important that members who are both infected and affected by HIV can join their group.

“This club has taken aboard a lot people, it did not discriminate whether one was ill or not ill, and by that I mean those who are infected with the virus. It has brought everyone together.” Saving and lending group member

The idea that both HIV negative and positive people should ‘join hands’ and work alongside each other was seen by a number of groups as a strategy to normalise HIV.

All community groups participating in this study provided their members with a sense of support and empowerment. Although some groups did not explicitly talk about HIV, most groups, through dialogue and reflection on the impact of HIV, provided members with the confidence to act on the HIV knowledge they received, as well as fostered a sense of responsibility to be active agents in the HIV response.

“Before I became part of the group before going for training on HIV/AIDS, before getting blood tested, I was afraid of it, blood testing. If I go for a blood test and be told that I have AIDS I will not cope. But we became part of the [co-operative] group and we discussed, I was taught and I began to know that this thing is there and is real, and when you have tested your blood, and you are diagnosed, you take your tablets, you live and last with your family until it grows. I realized that being part of the group is good because if I was alone I was not going to have the knowledge that I am supposed to go and get tested, I am supposed to learn, so that I can teach others about how good it is to get tested, how good knowing your status is.” Cooperative member

The community groups were also spaces where members were able to identify other community members who live positively, ‘done well’ in changing their lifestyle to avoid HIV, or to adhere to ART, and can act as role models.

“We usually get the messages on how one should behave in order to avoid HIV. Sometimes you hear that, ‘no it is difficult to control yourselves until you get married’, but then in this group we get a testimony of someone saying, ‘I have controlled myself now look the white veil is covering my face, right’. So we also have the desire that we want to be like them.” Youth group member

All the community groups provide members with the opportunity to develop close bonds with people outside their lineages and with people who share similar circumstances. For many members, the groups were effectively safe social spaces where they could come out and share their HIV status with other group members. This however was not the case for members of the farmer’s association, which was solely made up of men, and where group dynamics were driven by notions of masculinity that avoid talking about HIV. Yet, for the majority of the groups, it was a space where community members could talk openly about HIV and share information that makes them better equipped to either prevent HIV or adhere to their treatment.

“Joining this club has made me stay happy all the time and I am free from stress. If I’m stressed when I leave home, when I get here and the others ask me why I am so sad and I tell them the cause of my unhappiness, they comfort me and tell me all sorts of things to cheer me up. They tell me not to worry and that I will live to be a hundred years old. I am more confident now, in the past I used to be too embarrassed to be seen carrying my card that I use to get pills [ART] from the clinic but now I am so liberated that I do not hide.” HIV-positive member of savings and lending club

As the above quote also illustrates, community groups were also an important source of psychosocial support for the members themselves. The groups provided members with a space to experience joy and a sense of security from life stressors. In addition to being beneficial for the group members themselves, all groups, were committed to responding to needs of vulnerable community members – with the farmer’s association and the burial society groups less likely to do so with a HIV focus. There seemed to be a link between the sense of solidarity that the groups fostered and their desire to help people outside the group.

“Being part of a group has changed me because now I am able to go and fetch water for sick people and if I visit them and I see that they do not have soap and I have then I can cut a piece for them too, so that they may be able to bath.” Burial society member

Members of an AIDS support group, in particular, spoke about their devotion to reduce HIV transmission and to get people tested for HIV through encouragement and being open about their positive status.

“Our main aim was that we thought that if we start a group as people living with HIV we can try and reduce the HIV prevalence in our community and we seek to disseminate information in light of HIV/AIDS to those who are not in these groups, we try and tell them that if they have not yet been tested for HIV then they should go and get tested and should not hide their HIV status because having HIV does not mean that it is the end of life but it is actually the beginning of a new and better life.” AIDS support group member

Other community group members also spoke about their contribution to home-based care, both through more organised activities in collaboration with NGOs and through informal arrangements through the care and support of neighbours.

“We have come to realise that there are some who are ill and not able to support themselves. As women we go to them and do “something” for them, we fetch water, help them by waxing the floors or just do something for them that will help them after we leave. Even though they might have children who are able to do that for them, it will seem like we are “abusing” the children if we leave them do all those chores when, as a group we can assist with doing those chores.” Savings and lending group member

In summary, many community groups, in addition to their primary purpose of, for example, bringing community members together for life skills training, savings and lending, small scale farming or football, were, through dialogue, encouraging their members to engage with HIV, both to mitigate the impact of HIV on themselves and other members of their groups, but also to community members at large.

### Networks and partnerships

As the aforementioned observations allude to, collectivism, group formation and social action are key strategies to the indigenous HIV response in Matabeleland South. But what are the characteristics of these networks and partnerships in enabling community responses to HIV prevention, mitigation and care?

As described earlier, many parts of Matabeleland South are desolate, making it difficult for HIV-affected households to engage in subsistence farming and make ends meet. The difficult environment affects everyone, making it difficult for well-wishers to share the little they have. Even though community members form groups and have the motivation to support HIV-affected households, there is a limit to what they can practically do for community members living in difficult circumstances. For that reason, there was an overwhelming consensus that Matabeleland South is in urgent need for externally resourced organisations to come to the area and collaborate with the many community groups that make up the social landscape. Community group members argued that they had the motivation to support HIV-affected households, but argued that they lacked the resources to provide meaningful support. In summary, they argued that ‘real’ support is urgently needed.

“What I have noted is that we are not able to provide financial assistance. We are able to fetch water and bring an occasional meal but in some instances what is really required is money to buy food that will help the patient to maintain a healthy diet, or maybe the person has children and they need money to go to school.” Farmers group member

As mentioned earlier, Matobo District has got a number of active NGOs. These and other externally-resourced organisations were reported to fund HIV education programmes, water irrigation and farming programmes, community capacity building programmes and orphan care and support.

“The aid that that we get from NGOs that look to support people living with HIV is much appreciated. We feel taken care of and loved and we know that we are not alone in this battle that we are fighting.” AIDS support group member

Despite their presence, a number of the groups spoke about their limited support and only a few groups reported any kind of partnership with an externally-resourced organisation. There was a general consensus that the demand for external resources exceeds the supply and a number of pleas for support were conveyed through the interviews.

“The majority of us are facing many difficulties that is why we have joined the support group to support each other. Just now we have collected our small contributions but they will not take us very far. We need a “donor” that will sustain us and uplift us so that we will be able to fulfil the objectives that we have set for ourselves.” Savings and lending group member

As is clear from the above findings, the community group members believed that only by working together could they respond effectively to the epidemic. Working together in groups is therefore seen as a prerequisite for supporting HIV-affected households. Group formations and membership were also seen as a pathway to attract donor attention and funding. There was agreement across all the groups that groups and active community members are more likely to collaborate with and benefit from externally-resourced organisations. Many of the respondents hoped that their active participation in a group and implementing activities for themselves and HIV-affected households would be recognised by externally-resourced organisations that would subsequently use the community groups as an entry point for the delivery of support services. The participants gave a number of examples of where this had happened in the past, illustrating the relevance of this hope.

“I understood and came to terms with the fact that HIV/AIDS is here to stay. I also realised that being a member of a group could make me play a role in the fight against HIV/AIDS. Since I had it too there was need for me to think of ways that could help me live with it, so I had to think of how and doing what. So they had told us before that if you are a group it is easier to get assistance from outside, which is when we decided to form am group.” AIDS support group member

Furthermore, there was a widely-held belief that externally-resourced organisations had to operate through local structures in the delivery of aid and HIV services. So, for externally-resourced organisations to get the buy-in and be permitted to operate in a ward, approval from local chiefs, village elders and community group members was seen as necessary.

“It used to be difficult because back then we would just see the organisation come and just do what they want but now the community plays a role in determining what they get. We hold meetings as villages to determine what people need and who gets what first.” AIDS support group member

This gave the community group members a tremendous sense of control and ownership when working in collaboration with an externally-resourced organisation in the delivery of services. The attractiveness of community groups to NGOs, as well as the level of control community groups have in administering aid if partnering with an externally resourced organisation, may have contributed to the motivation of some community members to either join or establish a community group.

Although many of the community group members were keen to collaborate with externally-resourced organisations, there was a caveat to their enthusiasm. A number of participants expressed concern over externally-resourced organisations bypassing them and not consulting community members about their needs. It was argued that that NGOs draw on simplistic understandings of who is deserving of aid - resulting in unfair distribution.

“Sometimes the orphans that are under the care of the guardians who are part of the farmers group that is practising irrigation are denied assistance simply because the guardians are working at the irrigation. This does not go well with us because it is discriminatory and it hurts us to think that these children are also orphans but they are not getting any assistance.” Farmers association group member

NGOs were also said to have a simplistic understanding of local needs and to not approach development holistically. Whilst there was an appreciation of the support that externally-resourced organisations provide, it was seen as inadequate compared to need.

“Sometimes when the NGO comes with the intention of providing support, it is meant for a small number of people but the village is big and their number is small, so the majority of the people will be left behind and we are left with the task of feeding the people, but we do not have the resources.” Savings and lending group member

These findings indicate that community groups are hugely important in the local response to HIV and that these groups have the motivation and capacity to do more if working together with externally-resourced organisations.

### Community strengths and resources

It has long been recognised that rural communities in Zimbabwe have a ‘portfolio of assets’ that help them deal with hardship and transform livelihoods. But what are some of the latent community strengths and resources that can be drawn upon in HIV prevention, mitigation and care efforts in this context?

Improvements in access to ART in Matobo District have contributed to a normalisation of HIV. It is fair to say that improved HIV services, referred to as close access to health facilities that offer ART, have changed the social landscape regarding HIV. The fact that most people have been affected by HIV one way or the other and now have good knowledge about HIV transmission has demystified HIV and contributed to a normalisation of HIV. This, coupled with the understanding that contracting HIV is no longer a death sentence, has contributed to a gradual openness and acceptance of HIV – also amongst members of the farmer’s association.

“Nowadays there is a big difference because back then, it was not easy at all to talk about the disease. It was scary to talk about the disease because we were very afraid of it. These days we talk freely about it and we share advice and information on how to deal with the disease. It is becoming easier to talk about it but back then it would be very difficult for me to talk about HIV to anyone that I suspected to be infected by the virus.” Farmer’s association group member

Many community members argued that HIV could now be compared to other common chronic diseases such as diabetes. This has not only contributed to a perception that stigma is on the decrease, diminishing the acceptability of stigmatising attitudes and discrimination. As such, all participants spoke about the unacceptability of stigmatising people living with or affected by HIV and expressed their condemnation of anyone stigmatising those affected by the disease.

“Life has changed a lot for PLHIV because people used to discriminate against people living with HIV/AIDS but now this is unacceptable. PLHIV are accepted, very few people will laugh at you, and people now feel pity.” AIDS support group member

This normalisation of HIV has undoubtedly made it easier for those infected and affected by HIV to negotiate access for support from their social networks. A key observation from the interviews is that the local context in which these interviews were conducted is characterised by an ethic of care and assistance.

“My pride about this community is in that I think we are united in the way we operate. We love doing whatever we will be doing to support each other, helping each other all the time.” Cooperative group member

People had a sense of understanding of the hardship endured by some people and an acceptance that one day they might be in a similar situation. This understanding contributed to a sense of collective solidarity from which care and support hinges. It was evident throughout the interviews that there is a strong commitment to ‘do good’ and help those in need of support. This commitment was sometimes sparked by religion and ‘God’s wish’, but, for most of the time, it was talked about as an act of anticipated reciprocity.

“Caring for a child who is not yours is good because when they grow up, and if you treated them well, you will end up living well. That child will acknowledge you so much, and say ‘you raised me to become a certain person, you educated’. This child will eventually raise you and remove you from some of the difficult circumstances you were in.” Women’s group member

It was believed that, in showing compassion for your fellow community members, favours and support would be reciprocated if you find yourself in a situation where you need help. All of this contributes to a social norm and expectation to provide care and support for vulnerable community members.

Against this background, numerous examples emerged of how community members close to those sick took initiative to provide care and support. Children, for example, were repeatedly referred to as the primary carers of their HIV-infected parents. Children not only carried out nursing duties, such as feeding their parents and administering medicines, they also sustained their livelihoods through income generation and the fetching of firewood and water. One burial society member describes the social value of children as a source of caregiving.

“Having children, children are your future, as it is if I do not have children I will not live. If it happens that I fall ill, a child will nurse me, if I stroke and I cannot stand up, or become blind, they will help me. So if you have children you are proud because they are your future they will take care of you when you are no longer able to do it on your own.” Burial society group member

Neighbours and close relatives were also reported to support those who were ill as well as their children. It was common for neighbours to help out with the fetching of water and firewood as well as sharing their cooked food with the HIV-affected family.

### Obstacles and barriers are acknowledged and considered in responses to HIV

The community groups faced a number of obstacles and barriers in their efforts to meet group goals. These barriers can, if undermining group efforts, be demotivating and prevent people from joining community groups. To optimise the impact of group memberships, there is a need to recognise and address contextual obstacles and barriers. What are some of the more common obstacles and barriers to the community response to HIV in Matabeleland South?

The lack of rain, the long walking distances to the nearest water sources combined with infertile and rocky soils make many parts of Matabeleland South inhospitable and unsuitable for subsistence farming. As one youth group member indicate, the risk of drought was frequently mentioned by our respondents.

“What is bad about this place is that we have rainfall problems, so food thus becomes scarce if we are not assisted by the NGOs you may end up hearing about dead corpses being found in “Mat South”, so this place has drought.” Youth group member

HIV-affected families, therefore, often experience food insecurity, undermining children’s concentration in school and the efficacy of ART for those living with HIV. This, coupled with poverty, makes HIV-affected households very vulnerable and in need of food aid and nutritional garden projects.

“Our biggest challenge here is that of hunger, so we should really talk a lot about that because if you take that pill on an empty stomach by 9 you will be feeling dizzy and if you look at the granary there will be nothing for you to cook so that you can eat. We also have children and they have nothing and yet they need clothes and blankets.” AIDS support group member

Although this has been recognised by NGOs like World Vision, their food distribution programme for PLHIV is limited and does not reach everyone in need of food. Poverty also puts a strain on households who have agreed to foster orphaned children. Many guardians are therefore unable to provide adequate care and support for orphaned children.

“The challenge that I’m facing is that I'm taking care of my deceased brothers’ two orphans. I am no longer able to pay the school fees for them. One of the orphans is in Form Two and she might drop out of school this year because we have run out of options, the other one is in grade five. As you can see I am very old and I'm no longer able to do much and I have nowhere to send these orphans for help.” Farmers group members

Again, whilst some NGOs and programmes like the Basic Education Assistance Module (BEAM) have provided support for orphaned and HIV-affected children, it is argued by the study participants that they only reach a fraction of those in need. In essence, poverty was said to undermine the well-being of, and responses to, HIV affected and infected community members.

On a few occasions, macro-level influences were said to inhibit a conducive environment for the HIV response. Although the majority of respondents spoke about the promising opportunities of churches, some respondents said there was still a long way to go for churches to be fully supportive. Examples were given of churches that allow polygamy and encourage men to multiple wives. Respondents also said that few HIV infected people dared to come out in the open and declare to their congregation that they are HIV positive. For some of the respondents, this is an indicator of the intrinsic values of the Church and illustrates that religion continues to be a barrier in the response to HIV.

“I go to church at ZCC; at ZCC you will not find AIDS education. They cannot explain or tell you and be direct about HIV, they go around it and say it is obscene.” Women’s group member

Also at a macro-level, a few respondents spoke about how political turmoil and the change in currency had a devastating impact on their livelihoods. Water became unaffordable and groups relying on water for irrigation of their collective farms, as well as savings and lending groups, were suddenly unable to work towards their objectives.

“The irrigation was viable before the multicurrency economy but everything has since changed and the water charges have gone up. The money that we are making is all going towards the electricity and water charges. We are labouring in vain and can no longer provide for the orphans in our care. Those are the challenges that we are facing.” Farmers group member

Concerns were also raised about the poor infrastructure of the province, particularly in relation to the lack of water pumps and irrigation systems. A few people expressed the opinion that health facilities were too far away.

At a symbolic and cultural level, fear of HIV and AIDS continues to prevent some people and men in particular, from seeking support and treatment. When asked about gender differences in HIV service uptake, there was widespread agreement that men – because of their commitment to local understandings of what it means to be a real man – are much less likely to access HIV services.

“Men do not want to open up and admit that they are living with HIV. They only do that when the wife gets pregnant and tells the husband that the hospital staff want them to visit the hospital together or when he gets seriously ill and we visit and advise him to go get tested that is the only time maybe when they can go get tested otherwise they do not want” AIDS support group member

The fear of being associated with HIV prevented some people from asking for help. Relatedly, and reflecting the continued presence of stigma, some people would try very hard to hide their HIV status, making it difficult for community groups to identify those in need of help.

“We have a wish to help the people, and to know that it is they suffer from but most of them do not tell us their status. And also if you try to help them, those at home where they are staying will think that you think they are poor, and they may not want you to behave as if you are interfering into their lives.” Cooperative member

It is clear from this section that many challenges persist for community groups to respond effectively to the HIV epidemic.

## Discussion

The aim of this study was to explore the role of community groups in the HIV response. What emerged from our findings is that community members in Matobo – as a strategy to cope with hardship – cluster together into groups and social networks upon whom they can rely for care and support. The social networks span from close knit ties between neighbours and extended family to more organised community groups and right up to partnerships between community groups and externally-resourced organisations, such as NGOs and local government departments.

Community groups and networks do play a critical and enabling role in framing people’s response, both individually and collectively, to the HIV epidemic. Many groups, although not all, are active in HIV management activities. They mobilise community volunteers to act as community health workers or adherence support workers for people living with HIV. Community groups and networks not only support people living with HIV, they also provide members, many of whom are HIV affected themselves, with a safety net in times of hardship – both for emotional support and to access more material support from co-members. Many of our informants spoke about the need for community groups and networks to be linked with more resourceful organisations, enabling them to more effectively respond to HIV. They argued that community groups, in virtue of their knowledge and use of local strengths and resources, were in an ideal position to appropriate HIV service programmes. As such, community groups provide an entry point for international organisations seeking to support those affected by HIV.

They did however also discuss some of the many obstacles to an HIV response. In this context, the land is rugged, which coupled with infrequent rain and drought, made subsistence farming difficult for everyone – contributing to poverty and undermining local responses to HIV. Stigma, men’s fear of HIV and poor service delivery was also said to undermine the kind of support offered to those affected by HIV.

It is clear from our findings that community groups in this context are critical to the HIV response, yet have the potential to do so much more if working in partnership with more resourceful organisations. To improve the effectiveness of HIV programmes and the uptake of services, future HIV programming needs to embrace and encourage community participation and involvement in the HIV response, drawing sensitively on local resources and strengths, social networks and support strategies, whilst also being cognisant of local challenges and obstacles to the community response. This calls for collaborative partnerships between community groups and external change agents that are characterised by trust and transcend discrepancies in values, interests, knowledge and power. This is a notoriously difficult task and there is a risk that the partnerships may inadvertently be undermining local efforts to cope with the epidemic. In Rwanda for example, Thurman and colleagues [19] found that ill-conceived NGO services can fragment local community responses by taking over the perceived responsibility for care of children affected by HIV. So although community groups are key players in the apparatus facilitating a more effective HIV response, it remains a challenge to work out how best to establish collaborative partnerships between actors that do not unintentionally de-rail local responses. A promising method pertains to community-based capital cash transfers, where community groups are invited to draw up action plans that reflect local struggles and receive community-based cash grants to implement them. This might include setting up social enterprises for the support of orphaned and vulnerable children [20,21].

## Conclusion

The Investment Framework is seeking a more effective response to HIV and calls for programmatic strategies that can act as ‘critical enablers’ for improved HIV services access and uptake. Although the Framework acknowledges the importance of the community in the HIV response, little has been done to spell out how communities can get involved in the HIV response, let alone their ‘behind the scenes’ support. This paper has highlighted the active participation of informal community groups and networks in the HIV response, suggesting that community groups and networks are ‘critical enablers’ to the HIV response. As much as programmatic strategies and efficacious technologies play a central role in the HIV response, our findings suggest that community members, through networks and groups, carry the bulk of the HIV burden and need to be supported in their efforts. There is an urgent need to for greater synergy and collaboration between local community groups and networks, the health services and other external HIV service delivery sectors.

## Competing interests

The authors declare that they have no competing interests.

## Authors’ contributions

All authors were involved in ongoing discussions of the study design. MS managed the data set, conducted the data analysis and wrote the first draft of the paper. SM conducted the interviews. CN managed the fieldwork and compliance to research protocols. SG and CC were the principle investigators of the Community Response Project. All authors read and approved the final manuscript.

## Pre-publication history

The pre-publication history for this paper can be accessed here:

http://www.biomedcentral.com/1472-6963/13/195/prepub
